# Dynamic changes of alkaline phosphatase are strongly associated with PSA-decline and predict best clinical benefit earlier than PSA-changes under therapy with abiraterone acetate in bone metastatic castration resistant prostate cancer

**DOI:** 10.1186/s12885-016-2260-y

**Published:** 2016-03-14

**Authors:** Phillip Mikah, Laura-Maria Krabbe, Okyaz Eminaga, Edwin Herrmann, Philipp Papavassilis, Reemt Hinkelammert, Axel Semjonow, Andres-Jan Schrader, Martin Boegemann

**Affiliations:** Department of Urology, Muenster University Medical Center, Albert-Schweitzer-Campus 1, GB A1, D-48149 Muenster, Germany; Department of Urology, Cologne University Medical Center, Kerpener Strasse 62, GB Nr. 9, D-50937 Cologne, Germany

**Keywords:** mCRPC, Surrogate biomarker, Abiraterone acetate, Outcomes, Prostate cancer

## Abstract

**Background:**

Significant progress in treatment of metastatic castration resistant prostate cancer (mCRPC) has been made. Biomarkers to tailor therapy are scarce. To facilitate decision-making we evaluated dynamic changes of alkaline phosphatase (ALP), lactate dehydrogenase (LDH) and prostate specific antigen (PSA) under therapy with Abiraterone.

**Methods:**

Men with bone mCRPC (bmCRPC) on Abiraterone 12/2009-01/2014 were analyzed. Dynamic ALP-, LDH- and PSA-changes were analyzed as predictors of best clinical benefit and overall survival (OS) with logistic-regression, Cox-regression and Kaplan-Meier-analysis.

**Results:**

Thirty-nine pre- and 45 post-chemotherapy patients with a median follow up of 14.0 months were analyzed. ALP-Bouncing can be observed very early during therapy with Abiraterone. ALP-Bouncing is defined as rapidly rising ALP-levels independent of baseline ALP during the first 2–4 weeks of Abiraterone-therapy with subsequent equally marked decline to pretreatment levels or better within 8 weeks of therapy, preceding potentially delayed PSA-decline. In univariate analysis failure of PSA-reduction ≥50 % and failure of ALP-Bouncing were the strongest predictors of progressive disease (*p* = 0.003 and 0.021). Rising ALP at 12 weeks, no PSA-reduction ≥50 % and no ALP-Bouncing were strongest predictors of poor OS, (all *p* < 0.001). Kaplan-Meier-analysis showed worse OS for rising ALP at 12 weeks, no PSA-reduction ≥50 % and no ALP-Bouncing (*p* < 0.001). In subgroup-analysis of oligosymptomatic patients all parameters remained significant predictors of poor OS, with no PSA-reduction ≥50 % and rising ALP at 12 weeks being the strongest (*p* < 0.001). In multivariate analysis PSA-reduction ≥50 % remained an independent predictor of OS for the whole cohort and for the oligosymptomatic subgroup (both *p* = 0.014). No patient with ALP-Bouncing had PD for best clinical benefit. Patients with rising ALP at 12 weeks had no further benefit of Abiraterone.

**Conclusions:**

Dynamic changes of ALP, LDH and PSA during Abiraterone-therapy are associated with best clinical benefit and OS in bmCRPC. ALP-Bouncing occurring earlier than PSA-changes as well as prior to equivocal imaging results and rising ALP at 12 weeks under Abiraterone may help to decide whether to discontinue Abiraterone. An external validation of these findings on a prospective cohort is planned.

**Electronic supplementary material:**

The online version of this article (doi:10.1186/s12885-016-2260-y) contains supplementary material, which is available to authorized users.

## Background

During the past 10 years significant progress has been achieved in treatment of metastatic castration resistant prostate cancer (mCRPC). Several drugs with potential to extend overall survival (OS) have been approved, some of which harbor fairly little toxicity [1–9]. Amongst these Abiraterone acetate is available in pre- and post-chemotherapy setting and has become a broadly accepted standard of care. A major challenge for clinicians, researchers and indirectly for reimbursers is the measurement of treatment success due to lack of reliable, validated and easily available biomarkers for predicting outcomes and clinically relevant endpoints for modern therapies. Therefore, the decision for or against a drug out of an increasing repertory, therapy monitoring while on the drug and decision-making on continuation of therapy is delicate.

Several biomarkers have previously been described including prostate specific antigen (PSA), circulating tumor cells (CTC), alkaline phosphatase (ALP) and lactate dehydrogenase (LDH) [10–18]. However, these markers are not always straightforward or interpretable beyond doubt. PSA can rise during the first 3–6 months of therapy before declining [10, 18, 19]. Baseline CTC-enumeration and changes during therapy have been shown to be surrogate for survival endpoints [15], but lack broad availability and display limited accuracy with currently available kits [11, 16]. LDH is used in a multitude of cancers and baseline-level has been found to be surrogate for OS before Abiraterone-therapy [15] but is relatively unspecific. ALP has prognostic potential in mCRPC treated with Docetaxel and Radium-223 [12, 14, 17] but changes during therapy have not yet been evaluated in depth. An interesting phenomenon, which we call “ALP-Bouncing”, seems to happen with some patients at a very early stage of antihormonal therapy in bone mCRPC (bmCRPC) followed by sometimes dramatic and prolonged response. In these patients ALP may rise to extreme levels during the first 2–4 weeks before eventually declining to pre-treatment levels or better. This ALP-bouncing does not last longer than 8 weeks after initiation of therapy and usually precedes PSA decline. However, this event does not occur in a uniform way. The phenomenon was first described in 1945 after bilateral orchiectomy of a bone-metastatic prostate cancer patient [13]. To our knowledge, the relevance for therapy monitoring has never been investigated before.

Altogether biomarkers in mCRPC are yet to be established in many ways to help in decision-making and to guide treatment algorithms. In this study we aimed to investigate the possible surrogate function of ALP alongside PSA and LDH in bmCRPC-patients.

## Methods

### Patients

After IRB-approval (Muenster University Medical Faculty), all patients with mCRPC presenting at the Department of Urology, Muenster University Medical Center between 12/2009-01/2014 who received Abiraterone were retrospectively reviewed to evaluate possible biomarkers and their dynamic changes as surrogates for best clinical benefit and OS during very early Abiraterone-therapy. All patients gave written informed consent before participating (Additional file 1).

The patients presented with confirmed mCRPC (defined by PCWG2-criteria [20]) in pre- or post-chemotherapy setting suitable for Abiraterone-treatment. For the pre-chemotherapy subpopulation patients had to be asymptomatic or oligo-symptomatic with no use of opiates and a pain level of ≤3 out of 10 on the numeric-rating-scale. For the post-chemotherapy subpopulation, progressive disease (PD) had to be evident either on or after chemotherapy. A total of 96 patients presented during the reviewed time frame. Three of these patients had received prior Enzalutamide. One patient with a secondary malignancy and one beforehand treated with Ketokonazole were excluded from final analyses. Since ALP is a bone metastasis dependent marker, patients without bone metastases were excluded from final analyses, resulting in 84 evaluable patients, 39 in pre- and 45 in post-chemotherapy setting. All patients in the analysis were either on a stable dose of an antiresorptive agent (i.e. Zoledronic acid or Denosumab) at least 3 months prior to start of Abiraterone and during the whole treatment phase or did not receive antiresorptive medication at all. Four (4.7 %) patients were treated within the pre-chemotherapy registration trial for abiraterone (COU-AA-302) [4]. These patients were systematically followed up with 12-weekly imaging even when no PD was suspected. All other patients were treated within standard of care.

Patients presented the day before start of Abiraterone-therapy to have blood drawn for baseline analysis, two and 4 weeks after initiation of therapy and 4 weekly thereafter. LDH-, ALP- and PSA-levels were immediately measured in the serum samples within routine analysis. Dynamic changes of LDH, ALP and PSA were documented. ALP-Bouncing was defined as rapid elevation above the upper normal limit (UNL) during the first 2–8 weeks of Abiraterone-treatment with an equally marked decline to pretreatment levels or better within the same interval.

CT-, MRI- or Cholin-PET-CT-scans of thorax, abdomen and pelvis were used for the evaluation of soft tissue metastases. Bone scans were done to acquire additional information on baseline bone metastases. Imaging was repeated during Abiraterone-therapy when clinically indicated and not in routine fashion. PD was defined by RECIST 1.1 criteria [21] for cross sectional imaging and by PCWG2 criteria for bone scans [20].

A physician (MB) with large expertise in the treatment of mCRPC assessed the current response status i.e. complete remission (CR), partial remission (PR), stable disease (SD) or PD at each visit. For defining disease progression, deterioration of general condition, worsening of pain and worsening laboratory constellations as well as imaging were taken into account. Patients were subdivided into two groups according to ALP-status (group-1: no ALP-bouncing, group-2: ALP-bouncing). The study was performed according to the STROBE-criteria (Additional file 2: STROBE statement).

### Statistical methods

The descriptive statistics are reported as the medians with 95 % confidence intervals (CI) or interquartile range (IQR) for continuous variables and as populations and frequencies for categorical variables. The χ2-test, Fisher’s exact-test, t-test or Mann–Whitney U-test were performed to determine the significance of the differences between categorical and continuous variables, respectively. Survival analysis was performed with Kaplan-Meier-analysis [22]. Univariate and multivariate analysis of the different biomarkers were done with binary logistic-regression and Cox-regression-models. For all patients for whom time-dependent variables were used within the Cox-regression-models the follow-up period was at least 12 weeks long. All reported *p*-values are two-sided and statistical significance was assumed as *p* ≤ 0.05. SPSS-Statistics V.22 (IBM Inc., Armonk, NY) was used for statistical assessment.

## Results

### Patient characteristics

The final cohort consisted of 39 men in pre- and 45 in post-chemotherapy setting. Median follow-up was 14.0 (IQR 10.0–18.0) months for the patients alive at time of analysis. The median time on Abiraterone therapy was 10.0 months (IQR 5.0–14.0) with 18 (21 %) patients with ongoing therapy at the time of analysis. For one patient the Abiraterone dose had to be halved due to elevation of transaminases after 4 weeks of therapy. For all other patients no dose modifications were necessary. Descriptive characteristics of the cohort are given in Table 1. The median age of the patients was 69 years (IQR: 62.3–76.0). Visceral metastases were present in 20 % at the beginning of Abiraterone. Unfavorable Gleason-Score of ≥8 at initial diagnosis was found in 60.8 %. Median baseline PSA-, ALP- and LDH-levels were 174 ng/ml, 155 U/l and 287 U/l, respectively.

**Table 1:** Characteristics of patients with bmCRPC on AA with or without ALP-Bouncing

Variable	all	no ALP-Bouncing	ALP-Bouncing	p
Patients [n], (%)	84	50 (60)	34 (40)	-
Age, median [years] (IQR)	69.0 (62.3–76.0)	68.0 (61.0–75.5)	70.5 (66.5–76.0)	0.304
Lnn. Metastases [n] (%)	45 (53.6)	27 (54.0)	18 (52.9)	0.924
Visceral Metastases [n] (%)	17 (20.2)	14 (28.0)	3 (8.8)	0.032
Pre CTX [n] (%)	39 (46.4)	24 (48.0)	15 (44.1)	0.726
Post CTX [n] (%)	45 (53.6)	26 (52.0)	19 (55.9)	0.726
Patients died [n] (%)	53 (63.1)	39 (78.0)	14 (41.2)	-
Antiresorptive therapy [n] (%)	56 (66.7)	32 (64.0)	24 (70.6)	0.530
Zoledronic acid [n] (%)	42 (50.0)	24 (48.0)	18 (52.9)	0.821
Denosumab [n] (%)	14 (16.7)	8 (16.0)	6 (17.6)	
Best clinical outcome [n] (%)				
CR	1 (1.2)	0 (0)	1 (2.9)	<0.001
PR	51 (60.7)	22 (44.0)	29 (85.3)	
SD	20 (23.8)	16 (32.0)	4 (11.8)	
PD	12 (14.3)	12 (24.0)	0 (0)	
ECOG (all) [n] (%)				
0	12 (14.3)	7 (14.0)	5 (14.7)	0.351
1	53 (63.1)	29 (58.0)	24 (70.6)	
2	29 (22.6)	14 (28.0)	5 (14.7)	
GS ≥ 8 [n] (%)^a^	45 (60.8)	29 (67.4)	16 (51.6)	0.169
PSA red. ≥ 50 % [n] (%)	47 (56.0)	19 (38.0)	28 (82.4)	<0.001
PSA red. ≥ 90 % [n] (%)	22 (26.2)	6 (12.0)	16 (47.1)	<0.001
Median PSA Baseline [ng/ml] (IQR)	174 (55–500)	105 (47–457)	151 (54–477)	0.824
Median LDH Baseline [U/l] (IQR)	287 (223–422)	290 (228–565)	252 (218–327)	0.125
Median ALP Baseline [U/l] (IQR)	155 (102–355)	171 (96–381)	154 (114–273)	0.895
ALP rising at 12 w AA [n] (%)	27 (33.3)	27 (57.4)	0 (0)	-
LDH BL > UNL [n] (%)	58 (71.6)	37 (77.1)	21 (63.6)	0.187
LDH normalization [n] (%)	23 (39.0)	10 (27.0)	13 (59.1)	0.015

^a^Patients for whom the GS-information was not available were excluded from GS analysis (*n* = 10)

**Fig. 1:**
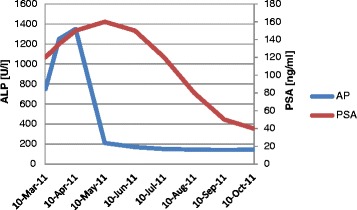
Rising PSA is a common phenomenon during the first 12 weeks of Abiraterone therapy followed by either continued rising PSA representing true disease progression or delayed PSA-decline representing response to therapy. ALP-Bouncing, occurring in 40 % of the studied patients with bmCRPC happens at a very early stage of antihormonal therapy and is defined as rapidly rising ALP-levels independent of baseline-ALP during the first 2–4 weeks of therapy with subsequent, equally marked, decline to pretreatment levels or better no later than 8 weeks after initiation of therapy. This can precede potentially delayed PSA-declines. This phenomenon does not occur in a uniform way

ALP-Bouncing is defined as rapidly rising ALP-levels independent of baseline ALP-level during the first 2–4 weeks of Abiratereone-therapy with subsequent equally marked decline to pretreatment levels or better within 8 weeks of therapy, preceding potentially delayed PSA-changes (Fig. 1). Patients with ALP-Bouncing had significantly less baseline visceral metastases (8.8 vs. 28.0 %, *p* = 0.032). Those with ALP-Bouncing had a higher percentage of CR, PR and SD (100 % vs. 76 %, *p* < 0.001) and LDH normalization (59.1 % vs. 27.0 %, *p* = 0.015) (Table 1).

**Fig. 2:**
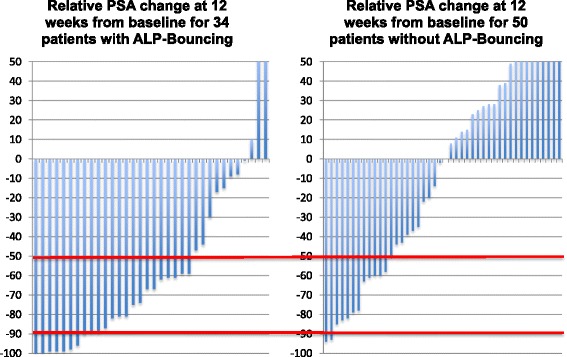
In the subpopulation of patients with ALP-Bouncing 68 % of patients had a PSA-decline of ≥50 % and 24 % even had a decline of ≥90 %. In patients without ALP-Bouncing only 30 % showed a PSA-decline of ≥50 % and only 5 % declined by ≥90 % (all *p* < 0.001)

In the subpopulation of patients with ALP-Bouncing 68 % had a PSA-decline of ≥50 % and 24 % a decline of ≥90 %. In patients without ALP-Bouncing 30 % showed a PSA-decline of ≥50 % and only 5 % declined by ≥90 % (all *p* < 0.001) (Fig. 2).

Antiresorptive therapy (Zoledronic acid or Denosumab) were well balanced in both the ALP-Bouncing- and non ALP-Bouncing-subgroups (70.6 and 64.0 %) (Table 1).

### Value of ALP, PSA and LDH as prognostic markers of clinical benefit

In univariate analyses of parameters for best clinical benefit (defined as PD vs. CR/PR/SD) presence of visceral metastases, no ALP-Bouncing, no PSA-reduction of ≥50 % and rising ALP at 12 weeks of Abiraterone-therapy showed a statistically significant higher risk of PD (Table 2). No PSA-reduction of ≥50 % and no ALP-Bouncing were the strongest predictors of PD (Odds ratio: 24.9, 95%CI: 3.1–202.1, *p* = 0.003 and 11.6, 95%CI: 1.4–93.5; *p* = 0.021, respectively)).

**Table 2:** Univariate analyses of biomarkers for progressive disease as best clinical benefit in bmCRPC on Abiraterone-therapy

Variable	OR (95 % CI)	p
PSA decline ≥ 50 % no vs. yes	24.9 (3.1–202.1)	0.003
No-Bouncing vs. ALP-Bouncing	11.6 (1.4–93.5)	0.021
ALP rising after 12 w yes vs. no	6.3 (1.7–22.8)	0.006
Visceral Mets. yes vs. no	4.0 (1.2–13.9)	0.028
ECOG 2 vs. 0–1	3.3 (1.0–11.1)	0.055
LDH normalization no vs. yes	3.0 (0.6–15.6)	0.192
LDH BL > UNL yes vs. no	2.2 (0.4–10.9)	0.338
ALP BL > UNL yes vs. no	1.3 (0.4–4.3)	0.698
AA post-CTX vs. pre-CTX	1.2 (0.4–3.7)	0.769
Lnn. Mets. yes vs. no	1.2 (0.4–3.8)	0.769
GS ≥ 8 vs. GS < 8	1.2 (0.3–4.4)	0.835

Results of univariate analyses for OS are displayed in Table 3 and in Additional file 1: Table S1. No ALP-Bounce, worse eastern cooperative oncology group performance status (ECOG) (2 vs. 0–1), no PSA-reduction of ≥50 % or ≥90 %, rising ALP at 12 weeks of Abiraterone-therapy, baseline-LDH > UNL and failure of LDH-normalization under Abiraterone-therapy were significant predictors of worse OS, with rising ALP at 12 weeks of Abiraterone-therapy, no PSA-reduction of ≥50 % and no ALP-Bouncing being the strongest predictors (Hazard-ratio (HR): 4.9, 95%CI: 2.7–8.9, 4.2, 95%CI: 2.3–7.6 and 3.1, 95%CI: 1.7–5.7; all *p* < 0.001, respectively). In subgroup analysis of patients with asymptomatic or oligosymptomatic bmCRPC (ECOG 0–1) (results are given in Additional file 1: Table S1) all of these parameters remained significant predictors of poor OS. No PSA-reduction of ≥50 % and rising ALP at 12 weeks showed the highest level of significance (HR: 5.7, 95%CI: 2.8–11.7 and 4.3, 95%CI: 2.1–8.7; both *p* < 0.001).

**Table 3:** Univariate and multivariate analyses of significant biomarkers for OS in 84 bmCRPC-patients under Abiraterone-therapy

Univariate analysis			Multivariate analysis		
Variable	HR (95 % CI)	p	Variable	HR (95 % CI)	p
PSA decline ≥ 50 % no vs. yes	4.2 (2.3–7.6)	<0.001	PSA decline ≥ 50 % no vs. yes	2.8 (1.2–6.3)	0.016
No-Bouncing vs. ALP-Bouncing	3.1 (1.7–5.7)	<0.001	No-Bouncing vs. ALP-Bouncing	2.0 (0.7–5.7)	0.181
ALP rising after 12 w yes vs. no	4.9 (2.7–8.9)	<0.001	ALP rising after 12 w yes vs. no	1.5 (0.6–3.9)	0.412
ECOG 2 vs. 0–1	2.7 (1.4–5.0)	0.002	ECOG 2 vs. 0–1	1.4 (0.6–3.1)	0.387
PSA decline ≥ 90 % no vs. yes	3.0 (1.5–6.1)	0.003	-	-	-
LDH normalization no vs. yes	2.6 (1.3–5.0)	0.005	LDH normalization no vs. yes	1.8 (0.8–3.9)	0.143
LDH BL > UNL yes vs. no	2.1 (1.0–4.3)	0.048	LDH BL > UNL yes vs. no	1.2 (0.2–7.1)	0.826
Visceral Mets. yes vs. no	1.8 (0.9–3.3)	0.076	Visceral Mets. yes vs. no	1.0 (0.5–2.2)	0.076
AA post-CTX vs. pre-CTX	1.5 (0.8–2.7)	0.175	-	-	-
ALP BL > UNL yes vs. no	1.4 (0.8–2.5)	0.199	-	-	-
GS ≥ 8 vs. GS < 8	1.3 (0.7–2.4)	0.365	-	-	-
Lnn. Mets. yes vs. no	0.9 (0.6–1.6)	0.804	-	-	-

**Fig. 3:**
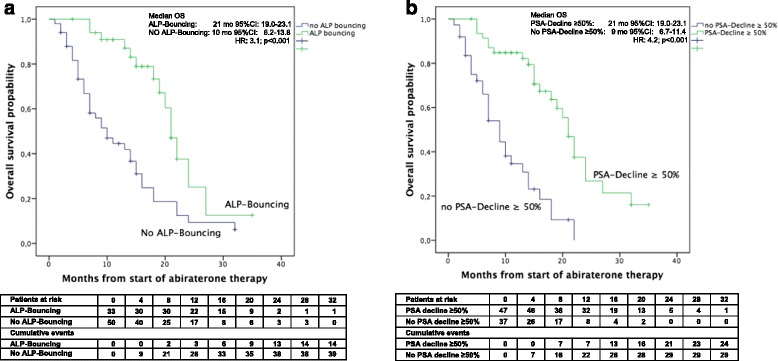
The Kaplan-Meier analysis showed significantly worse OS for no ALP-Bouncing (**a**) and no PSA-decline of ≥50 % (**b**). No ALP-Bouncing and no PSA-decline of ≥50 % were the strongest predictors of poor overall survival on Abiraterone therapy

Kaplan-Meier analyses for OS are shown in Fig. 3. All relevant markers identified in univariate analyses also showed significantly worse OS in Kaplan-Meier analyses, with rising ALP at 12 weeks of Abiraterone-therapy (median OS: 8 months, 95%CI: 5.9–10.0), no PSA-reduction of ≥50 % (median OS: 9 months, 95%CI: 6.7–11.4) and no ALP-Bouncing (median OS: 10 months, 95%CI: 6.2–13.8) being the strongest predictors (all *p* < 0.001).

### Multivariate evaluation of predictive biomarkers

In multivariate analyses for OS only no PSA-reduction of ≥50 % remained an independent significant predictor of OS (HR: 2.8, 95%CI: 1.2–6.3; *p* = 0.016) after adjusting for ALP-Bouncing, rising ALP at 12 weeks of Abiraterone-therapy, ECOG and LDH-normalization (Table 3). In multivariate analyses of the subgroup of asymptomatic and oligosymptomatic bmCRPC-patients no PSA decline of ≥50 % continued to be a significant predictor of poor OS (HR: 4.1, 95%CI: 1.3–12.9; *p* = 0.018) after adjusting for ALP-Bouncing, rising ALP at 12 weeks of Abiraterone-therapy and LDH-normalization (Additional file 1: Table S1).

Only 8 of 27 (30 %) patients with rising ALP at 12 weeks of Abiraterone without prior ALP-Bouncing had PR, 8 (30 %) had SD and 11 (40 %) had PD as best clinical benefit. In comparison, no patient with ALP-Bouncing had PD as best clinical benefit.

## Discussion

There are few biomarkers in mCRPC helping physicians in tailoring therapy. The utility of biomarkers depends on the clinical situation in which they are applied.

Especially in bmCRPC (80–90 % of all mCRPC-patients) early “bone-flare” in imaging is common under hormonal drugs like Abiraterone. This dilemma leads to many cases of misinterpretation, as apparent progression on imaging might be discordant with patients’ reports and clinical observation. This difficulty holds especially true in asymptomatic or oligo-symptomatic patients with no additional clinical information by change of symptoms [23].

PSA is most commonly used for therapy monitoring. However it may rise under therapy before declining. This PSA-flare can last as long as three or sometimes 6 months and mimic PD [19]. This may challenge the clinician whether to discontinue the drug, especially in patients with asymptomatic or oligosymptomatic disease [18]. Early rise of PSA can represent delayed response to therapy or even circulatory release of PSA due to tumor cell death and therefore harbor good prognosis [10]. The PCWG2 therefore recommends not stopping an individual therapy on the basis of early rising PSA alone. However, PCWG2 advises to document the efficacy of an individual therapy in clinical trials by determining the relative amount of patients with a PSA-decline of ≥50 % at 12 weeks [24]. In the registration trials for Abiraterone (COU-AA-302 and COU-AA-301) [4, 25], PSA-decline was no sole marker defining progressive disease. However, outside of clinical trial scenarios with the given problems of regularly delayed confirmation of dynamics of bone metastases on imaging, PSA is the only information available to the physicians to make decisions.

CTCs have been found to show changes earlier than PSA and no “flare” phenomenon has been reported. Dynamic changes in CTC-enumeration in early Abiraterone-therapy can serve as surrogate for response to treatment with potential translation into survival outcomes [15]. Early CTC-changes could help in steering therapy foremost in men with no or little symptoms. But CTCs have several disadvantages including high costs and limited routine availability to this point. Additionally CTCs measured with currently available assays display limited sensitivity [11, 16].

LDH is an unspecific biomarker in many tumor-types as well as mCRPC. Elevated or inclining LDH reflects higher tumor burden and/or high proliferation activity [26]. In the registration trial of Abiraterone for the post-chemotherapy setting baseline-LDH > UNL was prognostic for OS, therefore, a combination of LDH and CTCs has been proposed [15]. Rising LDH-levels under therapy indicate poor prognosis [27], and a normalization of a beforehand-elevated LDH might be predictive of response as well.

ALP has been shown to be prognostic in patients with mCRPC treated with Docetaxel and Radium-223 [12, 14, 17]. Thus serial measurements of ALP to discern dynamic changes may provide prognostic information in Abiraterone and other hormonal manipulations. To our knowledge this has not been studied before in relation to a specific antihormonal therapy. Especially ALP-Bouncing during very early therapy as described in this manuscript has not been reported previously.

With Abiraterone in the current treatment landscape being mostly used in the pre-chemotherapy setting in asymptomatic or oligosymptomatic patients the absence of symptoms brings along the physicians dilemma of not being able to determine the state of clinical response from the course of patients’ wellbeing alone. Lack of clinical symptoms with rising PSA can only be dealt with by imaging. Imaging itself, typically performed at 12 weeks of treatment, brings along a high rate of bone flares suggesting disease progression [23], leaving the physician in the dilemma of not knowing if continuation of a drug will be beneficial for the patient. Until now it is state of the art not to consider a sole PSA-rise a criterion of progress [20] and to continue medication until clinical progression occurs or repeated imaging several months later confirms progression. This approach harbors the possibility of unwillingly missing progression and continuing an inefficacious therapy. The individual patient might even lose time in which he could have been treated with other possibly life-prolonging drugs. This procrastination of switching therapy may lead to worsening of physical reserves for e.g. chemotherapy and render it in some cases impossible due to physical deterioration. The adherence to a non-efficacious therapy could also increase the preexisting financial burden for reimbursers.

Future biomarkers might help to improve customized therapy approaches in a growing landscape of treatment options. One possible marker was recently reported with the splice variant AR-V7 as a marker to predict response to subsequent therapy with Enzalutamide or Abiraterone before start of the drug [28]. A potential role of AR-V7 or other splice-variants in guiding Abiraterone-therapy remains to be established.

This study is limited by deficiencies inherent to a retrospective review. The cohort of 84 bmCRPC-patients is a very large number of consecutive patients for a single center study in this setting, however when subgrouping was performed the group size became relatively small. Also, patients in pre- and post-chemotherapy setting were grouped together, due to cohort size. In the COU-AA-302- and −301-trials radiographic progression-free survival was a primary endpoint [4, 25]. Per protocol up to 2 new bone metastases in the first bone scan after 12 weeks of abiraterone-therapy were primarily considered a potential bone flare and had to be confirmed with at least 2 additional bone metastases in a subsequent bone scan 8–12 weeks later. In our study systematic imaging was not performed 12-weekly. When PD was suspected imaging was performed for confirmation in most of the cases except in patients with unequivocal progression. Therefore, radiographic progression-free survival was not assessed. In our opinion this does not impair the results of our trial since very early ALP-dynamics are not meant to be parameters to define progression at a specified point in time but to predict the general outcome of Abiraterone-therapy much earlier than current imaging could.

Since antiresorptive therapy was well balanced between Zoledronic acid and Denosumab, when applied at all, and stable antiresorptive therapy had to be established within all patients analyzed, our results are not affected by these drugs with potential impact on ALP-levels.

Our study is the first to address dynamic changes of the easily available biomarkers ALP, LDH and PSA during very early Abiraterone-therapy in bmCRPC. Our results underline the importance of PSA-decline of ≥ 50 % as an important surrogate for best clinical benefit and OS as suggested by the PCWG2 [20]. Additionally, the presence of ALP-Bouncing in the first 2–8 weeks of therapy is a strong predictor for subsequent long lasting clinical response. In comparison to PSA with a relevant number of flares during the first 3–6 months of therapy, ALP gives earlier and straightforward information on the future course of disease in an important subset of patients. However, in our study ALP-Bouncing was not an independent predictor for OS. The reason for this is that ALP-Bouncing and PSA-decline of ≥ 50 % are not discrepant in a relevant number of cases, so for the given number of patients there are not enough differences to show significance in multivariate analysis for both variables. Nevertheless, our findings are very relevant for patients with non-concordant alterations. In our opinion, it appears to be safe to continue with Abiraterone whenever ALP-Bouncing has occurred even when PSA has not declined by ≥50 %. All patients who had rising ALP > UNL at 12 weeks of Abiraterone-therapy with no prior ALP-Bouncing had a bad course of disease when continued on Abiraterone even with ECOG 0–1. Therefore, from our perspective it might be equally reasonable to stop Abiraterone treatment in this constellation in carefully selected patients with bmCRPC. This could improve current practice of having to wait for repeat bone scans to confirm progression in asymptomatic or oligosymptomatic patients. However, prior to external validation of our findings PD should be proven by imaging before discontinuation of therapy.

These findings should be confirmed in other populations, preferably in prospective trials or at least in post-hoc analyses of large trial populations in which all these parameters were regularly assessed (e.g. COU-AA-302). A prospective validation is currently planned.

Despite these limitations the study is the first that relates to very early changes of an easily available biomarker showing potential to aid physicians to tailor therapy earlier than PSA and imaging in a difficult clinical situation.

## Conclusions

ALP-Bouncing is a promising biomarker which might aid at a very early stage of Abiraterone-therapy to safely continue or stop Abiraterone at 8–12 weeks in the described constellations of asymptomatic or mildly symptomatic patients with bmCRPC.

## Additional files

Additional file 1: STROBE statement. (DOC 97 kb) Additional file 2: Table S1. Univariate and multivariate analyses of significant biomarkers for OS in 65 bmCRPC-patients and ECOG 0-1 under Abiraterone-therapy. (DOCX 12 kb)

## Abbreviations

AA: abiraterone acetate
ALP: alkaline phosphatase
BL: baseline
bmCRPC: bone mCRPC
CI: confidence interval
CR: complete remission
CTC: circulating tumor cells
ECOG: eastern cooperative oncology group performance status
GS: Gleason score
HR: hazard ratio
IQR: interquartile range
LDH: lactate dehydrogenase
Lnn: lymph nodes
mCRPC: metastatic castration resistant prostate cancer
OS: overall survival
PD: progressive disease
PR: partial remission
PSA: prostate specific antigen
SD: stable disease
UNL: upper normal limit
