# Supplementary material for: Dynamic changes of alkaline phosphatase are strongly associated with PSA-decline and predict best clinical benefit earlier than PSA-changes under therapy with abiraterone acetate in bone metastatic castration resistant prostate cancer
Source: BMC Cancer. 2016 Mar 14;16:214. doi: 10.1186/s12885-016-2260-y (PMC4790058; doi:10.1186/s12885-016-2260-y)
Supplement: Additional file 1: — STROBE statement. (DOC 97 kb) [file 12885_2016_2260_MOESM1_ESM.doc]

Additional file 2

STROBE Statement—checklist of items that should be included in reports of observational studies

|  | | Item No | Recommendation |
| --- | --- | --- | --- |
| **Title and abstract** | | 1 | (*a*) Indicate the study’s design with a commonly used term in the title or the abstract:  Dynamic changes of alkaline phosphatase are strongly associated with PSA-decline and predict best clinical benefit earlier than PSA-changes under therapy with abiraterone acetate in bone metastatic castration resistant prostate cancer |
|  |  |  | (*b*) Provide in the abstract an informative and balanced summary of what was done and what was found  **Abstract**  **Background**  Significant progress in treatment of metastatic castration resistant prostate cancer (mCRPC) has been made. Biomarkers to tailor therapy are scarce. To facilitate decision-making we evaluated dynamic changes of alkaline phosphatase (ALP), lactate dehydrogenase (LDH) and prostate specific antigen (PSA) under therapy with Abiraterone.  **Methods**  Men with bone mCRPC (bmCRPC) on Abiraterone 12/2009-01/2014 were analyzed. Dynamic ALP-, LDH- and PSA-changes were analyzed as predictors of best clinical benefit and overall survival (OS) with logistic-regression, Cox-regression and Kaplan-Meier-analysis.  **Results**  Thirty-nine pre- and 45 post-chemotherapy patients with a median follow up of 14.0 months were analyzed. ALP-Bouncing can be observed very early during therapy with Abiraterone. ALP-Bouncing is defined as rapidly rising ALP-levels independent of baseline ALP during the first 2-4 weeks of Abiraterone-therapy with subsequent equally marked decline to pretreatment levels or better within 8 weeks of therapy, preceding potentially delayed PSA-decline. In univariate analysis failure of PSA-reduction ≥50% and failure of ALP-Bouncing were the strongest predictors of progressive disease (p=0.003 and 0.021). Rising ALP at 12 weeks, no PSA-reduction ≥50% and no ALP-Bouncing were strongest predictors of poor OS, (all p<0.001). Kaplan-Meier-analysis showed worse OS for rising ALP at 12 weeks, no PSA-reduction ≥50% and no ALP-Bouncing (p<0.001). In subgroup-analysis of oligosymptomatic patients all parameters remained significant predictors of poor OS, with no PSA-reduction ≥50% and rising ALP at 12 weeks being the strongest (p<0.001). In multivariate analysis PSA-reduction ≥50% remained an independent predictor of OS for the whole cohort and for the oligosymptomatic subgroup (both p=0.014). No patient with ALP-Bouncing had PD for best clinical benefit. Patients with rising ALP at 12 weeks had no further benefit of Abiraterone.  **Conclusions**  Dynamic changes of ALP, LDH and PSA during Abiraterone-therapy are associated with best clinical benefit and OS in bmCRPC. ALP-Bouncing occurring earlier than PSA-changes as well as prior to equivocal imaging results and rising ALP at 12 weeks under Abiraterone may help to decide whether to discontinue Abiraterone. An external validation of these findings on a prospective cohort is planned. |
| Introduction | | | |
| Background/rationale | | 2 | Explain the scientific background and rationale for the investigation being reported  Several new drugs have been approved to treat mCRPC in recent years, amongst others Abiraterone. However measurement of treatment success and decision making whether to stop or continue therapy is difficult during early therapy since the everyday used biomarker PSA often times provides non straight forward information. Additionally, imaging of bony metastases during the first 12 weeks of therapy can can feign disease progression although treatment response is in reality taking place. This bone flare phenomenon and true progression of bone metastases are hard to differentiate in asymptomatic patients. This leads to the current practice to continue treatment with Abiraterone even these patients not knowing if progression or remission is evident until the true nature of the event can be clarified with another staging 8-12 weeks later.  We discovered that ALP-Bouncing occurs earlier than PSA changes in patients with bone metastatic mCRPC and is associated with durable responses. |
| Objectives | | 3 | State specific objectives, including any prespecified hypotheses  In this study we aimed to investigate the possible surrogate function of ALP alongside PSA and LDH for best clinical benefit, progression-free survival and overall survival in bmCRPC-patients. |
| Methods | | | |
| Study design | | 4 | Present key elements of study design early in the paper  This is a retrospective cohort study on mCRPC patients treated with Abiraterone with or without ALP-Bouncing comparing ALP-Bouncing as a therapy control marker compared with PSA and LDH-changes. |
| Setting | | 5 | Describe the setting, locations, and relevant dates, including periods of recruitment, exposure, follow-up, and data collection  Setting: outpatient ward for urooncology, bone mCRPC  Location: University hospital Muenster, Germany, Department of Urology  Recruitment time: 12/2009-01/2014  Exposure: Median time of Abiraterone treatment: 10.0 months  Median follow-up: 14.0 months |
| Participants | | 6 | (*a*) *Cohort study*—Give the eligibility criteria, and the sources and methods of selection of participants.  All patients presenting with bone mCRPC and meeting the requirements for the lable of Abiraterone prior to or after Docetaxel chemotherapy were eligible.  Part of the patients were selected out of the patients at the University hospital in Muenster, Germany. The others were referred by general physicians or urologists at other sites.  Describe methods of follow-up  Dynamic changes of LDH, ALP and PSA were documented. ALP-Bouncing was defined as rapid elevation above the upper normal limit (UNL) during the first 2-8 weeks of Abiraterone-treatment with an equally marked decline to pretreatment levels or better within the same interval.  CT-, MRI- or Cholin-PET-CT-scans of thorax, abdomen and pelvis were used for the evaluation of soft tissue metastases. Bone scans were done to acquire additional information on baseline bone metastases. Imaging was repeated during Abiraterone-therapy when clinically indicated and not in routine fashion. PD was defined by RECIST 1.1 criteria for cross sectional imaging and by PCWG2 criteria for bone scans.  A physician with large expertise in the treatment of mCRPC assessed the current response status i.e. complete remission (CR), partial remission (PR), stable disease (SD) or PD at each visit. For defining disease progression, deterioration of general condition, worsening of pain and worsening laboratory constellations as well as imaging were taken into account.  Follow-up for survival was done with telephone calls. |
|  |  |  |  |
| Variables | | 7 | Clearly define all outcomes, exposures, predictors, potential confounders, and effect modifiers. Give diagnostic criteria, if applicable  Outcomes: Best clinical benefit, PFS, OS  Exposures: Abiraterone treatment  Prognosticators: ALP-Bouncing, PSA-Decline ≥50%, Rising ALP at 12 weeks, initially elevated LDH, LDH-normalization, ECOG, presence of visceral metastases, initially elevated ALP, presence of lymphonodal metastases, Gleason score < or ≥ 8, Abiraterone prior to or after Docetaxel |
| Data sources/ measurement | | 8* | For each variable of interest, give sources of data and details of methods of assessment (measurement). Describe comparability of assessment methods if there is more than one group  Laboratory biomarker: ALP, LDH, PSA  Imaging biomarker: metastases  Pathology biomarker: Gleason score  General condition: ECOG |
| Bias | | 9 | Describe any efforts to address potential sources of bias  All patients presenting with bone mCRPC and treated with Abiraterone were included in the study |
| Study size | | 10 | Explain how the study size was arrived at  84 patients were treated with Abiraterone and retrospectively analysed. |
| Quantitative variables | | 11 | Explain how quantitative variables were handled in the analyses. If applicable, describe which groupings were chosen and why  The descriptive statistics are reported as the medians with 95% confidence intervals or interquartile range for continuous variables and as populations and frequencies for categorical variables. The χ2-test, Fisher’s exact-test, t-test or Mann-Whitney U-test were performed to determine the significance of the differences between categorical and continuous variables, respectively. |
| Statistical methods | | 12 | (*a*) Describe all statistical methods, including those used to control for confounding |
|  |  |  | (*b*) Describe any methods used to examine subgroups and interactions |
|  |  |  | (*c*) Explain how missing data were addressed |
|  |  |  | (*d*) *Cohort study*—If applicable, explain how loss to follow-up was addressed  *Case-control study*—If applicable, explain how matching of cases and controls was addressed |
|  |  |  | (*e*) Describe any sensitivity analyses  The descriptive statistics are reported as the medians with 95% confidence intervals or interquartile range for continuous variables and as populations and frequencies for categorical variables. The χ2-test, Fisher’s exact-test, t-test or Mann-Whitney U-test were performed to determine the significance of the differences between categorical and continuous variables, respectively. Survival analysis was performed with Kaplan-Meier-analysis. Univariate and multivariate analysis of the different biomarkers were done with binary logistic-regression and Cox-regression-models. For all patients for whom time-dependent variables were used within the Cox-regression-models the follow-up period was at least 12 weeks long. All reported p-values are two-sided and statistical significance was assumed as p≤0.05.  Missing values (n=4 patients with no available Gleason-Score were censored from anlayses involving Gleason Scores) |
| Results | | | |
| Participants | 13* | (a) Report numbers of individuals at each stage of study—eg numbers potentially eligible, examined for eligibility, confirmed eligible, included in the study, completing follow-up, and analysed  84 patients with bone mCRPC | |
|  |  | (b) Give reasons for non-participation at each stage  none | |
|  |  | (c) Consider use of a flow diagram  N/A | |
| Descriptive data | 14* | (a) Give characteristics of study participants (eg demographic, clinical, social) and information on exposures and potential confounders | |
|  |  | (b) Indicate number of participants with missing data for each variable of interest | |
|  |  | (c) *Cohort study*—Summarise follow-up time (eg, average and total amount)  all included in tables or manuscript | |
| Outcome data | 15* | *Cohort study*—Report numbers of outcome events or summary measures over time  Done | |
|  |  |  |  |
|  |  |  |  |
| Main results | 16 | (*a*) Give unadjusted estimates and, if applicable, confounder-adjusted estimates and their precision (eg, 95% confidence interval). Make clear which confounders were adjusted for and why they were included | |
|  |  | (*b*) Report category boundaries when continuous variables were categorized | |
|  |  | (*c*) If relevant, consider translating estimates of relative risk into absolute risk for a meaningful time period  All done | |
| Other analyses | 17 | Report other analyses done—eg analyses of subgroups and interactions, and sensitivity analyses  Subgroup analysis for patients with no or little symptoms (ECOG 0-1) was done and details are given in the manuscript and in additional table 1. | |
| Discussion | | | |
| Key results | 18 | Summarise key results with reference to study objectives  done | |
| Limitations | 19 | Discuss limitations of the study, taking into account sources of potential bias or imprecision. Discuss both direction and magnitude of any potential bias  This study is limited by deficiencies inherent to a retrospective review. The cohort of 84 bmCRPC-patients is a very large number of consecutive patients for a single center study in this setting, however when subgrouping was performed the group size became relatively small. Also, patients in pre- and post-chemotherapy setting were grouped together, due to cohort size. In the COU-AA-302- and -301-trials radiographic progression-free survival was a primary endpoint. Per protocol up to 2 new bone metastases in the first bone scan after 12 weeks of abiraterone-therapy were primarily considered a potential bone flare and had to be confirmed with at least 2 additional bone metastases in a subsequent bone scan 8-12 weeks later. In our study systematic imaging was not performed 12-weekly. When PD was suspected imaging was performed for confirmation in most of the cases except in patients with unequivocal progression. Therefore, radiographic progression-free survival was not assessed. In our opinion this does not impair the results of our trial since very early ALP-dynamics are not meant to be parameters to define progression at a specified point in time but to predict the general outcome of Abiraterone-therapy much earlier than current imaging could | |
| Interpretation | 20 | Give a cautious overall interpretation of results considering objectives, limitations, multiplicity of analyses, results from similar studies, and other relevant evidence  ALP-Bouncing is a promising biomarker which might aid at a very early stage of Abiraterone-therapy to safely continue or stop Abiraterone at 8-12 weeks in the described constellations of asymptomatic or mildly symptomatic patients with bmCRPC. | |
| Generalisability | 21 | Discuss the generalisability (external validity) of the study results  Has been discussed | |
| Other information | | | |
| Funding | 22 | Give the source of funding and the role of the funders for the present study and, if applicable, for the original study on which the present article is based  No funding | |
