# Supplementary material for: Dynamic changes of alkaline phosphatase are strongly associated with PSA-decline and predict best clinical benefit earlier than PSA-changes under therapy with abiraterone acetate in bone metastatic castration resistant prostate cancer
Source: BMC Cancer. 2016 Mar 14;16:214. doi: 10.1186/s12885-016-2260-y (PMC4790058; doi:10.1186/s12885-016-2260-y)
Supplement: Additional file 2: Table S1. — Univariate and multivariate analyses of significant biomarkers for OS in 65 bmCRPC-patients and ECOG 0-1 under Abiraterone-therapy. (DOCX 12 kb) [file 12885_2016_2260_MOESM2_ESM.docx]

Additional file 1: Table S1: Univariate and multivariate analyses of significant biomarkers for OS in 65 bmCRPC-patients and ECOG 0-1 under Abiraterone-therapy.

| **Univariate analysis** | | | **Multivariate analysis** | | |
| --- | --- | --- | --- | --- | --- |
| **Variable** | HR (95% CI) | p | **Variable** | HR (95% CI) | p |
| **PSA decline ≥ 50% no vs. yes** | **5.7 (2.8-11.7)** | **< 0.001** | **PSA decline ≥ 50% no vs. yes** | **4.1 (1.3-12.9)** | **0.018** |
| **ALP rising after 12 w yes vs. no** | **4.3 (2.1-8.7)** | **< 0.001** | ALP rising after 12 w yes vs. no | 1.2 (0.4-3.7) | 0.711 |
| **PSA decline ≥ 90% no vs. yes** | **3.1 (1.3-7.0)** | **0.008** | - | - | - |
| **No-Bouncing vs. ALP-Bouncing** | **2.4 (1.2-4.9)** | **0.012** | No-Bouncing vs. ALP-Bouncing | 1.8 (0.6-5.2) | 0.281 |
| **LDH normalization no vs. yes** | **2.1 (1.0-4.4)** | **0.044** | LDH normalization no vs. yes | 1.4 (0.6-3.2) | 0.442 |
| **AA post-CTX vs. pre-CTX** | **2.1 (1.0-4.4)** | **0.046** | **-** | **-** | **-** |
| Visceral Mets. yes vs. no | 1.9 (0.9-4.0) | 0.113 | Visceral Mets. yes vs. no | 0.9 (0.3-2.4) | 0.813 |
| LDH BL > UNL yes vs. no | 1.8 (0.8-4.0) | 0.186 | - | - | - |
| ALP BL > UNL yes vs. no | 1.3 (0.7-2.5) | 0.412 | - | - | - |
| GS ≥ 8 vs. GS < 8 | 1.3 (0.7-2.7) | 0.427 | - | - | - |
| Lnn. Mets. yes vs. no | 0.9 (0.5-1.7) | 0.675 | - | - | - |
